# Impact of Long-Term Growth Hormone Replacement Therapy on Metabolic and Cardiovascular Parameters in Adult Growth Hormone Deficiency: Comparison Between Adult and Elderly Patients

**DOI:** 10.3389/fendo.2021.635983

**Published:** 2021-02-25

**Authors:** Elisabetta Scarano, Enrico Riccio, Teresa Somma, Rossana Arianna, Fiammetta Romano, Elea Di Benedetto, Giulia de Alteriis, Annamaria Colao, Carolina Di Somma

**Affiliations:** ^1^Dipartimento di Medicina Clinica e Chirurgia, Sezione di Endocrinologia, Università degli Studi di Napoli Federico II, Naples, Italy; ^2^Dipartimento di Neuroscienze e Scienze Riproduttive e Odontostomatologiche, Divisione di Neurochirurgia, Università degli Studi di Napoli Federico II, Naples, Italy

**Keywords:** growth hormone (GH) treatment, elderly, metabolism, cardiovascular, hypopituitarism

## Abstract

Growth hormone deficiency (GHD) in adults is due to a reduced growth hormone (GH) secretion by the anterior pituitary gland which leads to a well-known syndrome characterized by decreased cognitive function and quality of life (QoL), decreased bone mineral density (BMD), increased central adiposity with a reduction in lean body mass, decreased exercise tolerance, hyperlipidemia and increased predisposition to atherogenesis. Considering some similar features between aging and GHD, it was thought that the relative GH insufficiency of the elderly person could make an important contribution to the fragility of elderly. GH stimulation tests are able to differentiate GHD in elderly patients (EGHD) from the physiological reduction of GH secretion that occurs with aging. Although there is no evidence that rhGH replacement therapy increases the risk of developing Diabetes Mellitus (DM), reducing insulin sensitivity and inducing cardiac hypertrophy, long-term monitoring is, however, also mandatory in terms of glucose metabolism and cardiovascular measurements. In our experience comparing the impact of seven years of rhGH treatment on metabolic and cardiovascular parameters in GHD patients divided in two groups [adult (AGHD) and elderly (EGHD) GHD patients], effects on body composition are evident especially in AGHD, but not in EGHD patients. The improvements in lipid profile were sustained in all groups of patients, and they had a lower prevalence of dyslipidemia than the general population. The effects on glucose metabolism were conflicting, but approximately unchanged. The risk of DM type 2 is, however, probably increased in obese GHD adults with impaired glucose homeostasis at baseline, but the prevalence of DM in GHD is like that of the general population. The increases in glucose levels, BMI, and SBP in GHD negatively affected the prevalence of Metabolic Syndrome (MS) in the long term, especially in AGHD patients. Our results are in accordance to other long-term studies in which the effects on body composition and lipid profile are prominent.

## Introduction

Growth hormone deficiency (GHD) in adults is due to a reduced growth hormone (GH) secretion by the anterior pituitary gland which leads to a well-known syndrome characterized by decreased cognitive function and quality of life (QoL), decreased bone mineral density (BMD), increased central adiposity with a reduction in lean body mass, decreased exercise tolerance, hyperlipidemia, and increased predisposition to atherogenesis (1–3). For this reason, twenty years ago GH replacement therapy started to be used also in adults with GHD and not only in children with impaired growth. In fact, in 1989 initial studies on rhGH treatment in adult hypopituitary patients were published, with positive effects on the main features of the above-mentioned syndrome (4–9). On these bases, GH treatment was approved for Adult GHD (AGHD) in Europe in 1995 and in the United States (US) in 1996 (10). On this concern, elderly patients are a peculiar task: in these patients, whom we assist at a physiological reduction of GH secretion due to the advancing age, it is difficult to establish the right clinical threshold to start rhGH replacement therapy (11, 12). The reduction of GH/insulin like growth factor I (IGF-I) activity is considered to be one of the causes of catabolic process of normal aging and can partly explain the age-related variations in the bone metabolism, muscle mass, cardiovascular system, immune system and well-being, although sex steroids and malnutrition have an important role too. Considering some similar features between aging and GHD, it was thought that the relative GH insufficiency of elderly people could make an important contribution to the fragility of the elderly (13). GH stimulation tests are able to differentiate GHD in elderly patients (EGHD) from the physiological reduction of GH secretion that occurs with aging (11). A small number of randomized controlled trials have studied the effects of rhGH therapy in EGHD with no univocal data and no long period of treatment (14–16).

On these bases, the aim of our study was to evaluate the impact of seven years of rhGH treatment on metabolic and cardiovascular parameters in EGHD and AGHD. In addition, we evaluated the prevalence of dyslipidemia, type II diabetes mellitus (DM), metabolic syndrome (MS) according to IDF (MS-IDF) and ATP III (MS-ATPIII) criteria and arterial hypertension in GHD patients, and we compare it to prevalence of these comorbidities in a group of age-matched no GHD hypopituitary patients and in a group of age-matched controls.

## Materials and Methods

The study was approved by the local ethics committee and complied with the Declaration of Helsinki, in line with the Guidelines for Good Clinical Practice. All patients provided written informed consent before entering the study, with respect to study participation, and confidentiality statement of data collection according to the Italian privacy policy.

### Patients

In this study we evaluated 196 consecutive hypopituitary patients (125 GHD and 71 no GHD patients) followed at the outpatient clinic of the Department of Clinical Medicine and Surgery, Section of Endocrinology, “Federico II” University, Naples, Italy, from 1998 to 2010. We started recruiting patients in 1998 and ended in 2010, but we waited until 2017 to ensure that all patients had a 7-year follow up. From 2017 to nowadays we processed data and develop the manuscript.

The longest follow-up period that was available for our study population of adult patients was seven years. Seven years represent for GHD patients in treatment with rhGH a long-term follow-up. Therefore, to homogenize the sample the maximum follow-up period was set at seven years.

**Figure 1:**
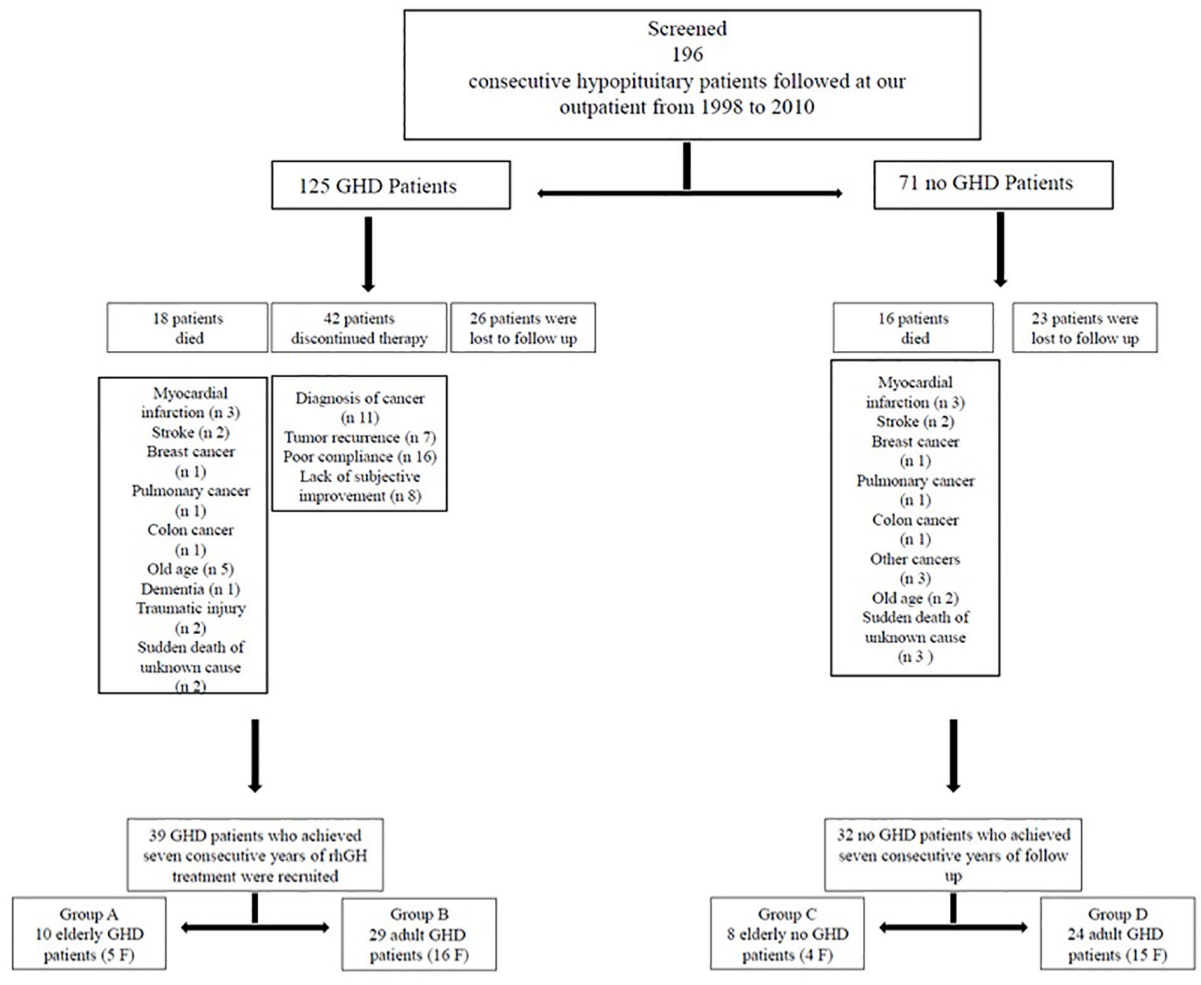
GHD and no GHD patients’ selection.

The following exclusion criteria were taken into account: 1) gpatients with rh-GH therapy discontinuation (more than 1 year); 2) patients who had missed visits more than three times. During the study period 34 patients died: myocardial infarction (n 6), stroke (n 4), breast cancer (n 2), pulmonary cancer (n 2), colon cancer (n 2), other cancers (n 3), old age (n 7), dementia (n 1), traumatic injury (n 2), and sudden death of unknown cause (n 5); 42 GHD patients discontinued therapy more than 1 year: diagnosis of cancer (n 11), tumor recurrence (n 7), poor compliance (n 16), lack of subjective improvement (n 8); 49 patients were lost to follow-up (**Figure 1**). So, we recruited 39 GHD patients (21 F, 18 M, mean age 48.38 ± 13.33 years, range 26–71 years) who achieved at least seven consecutive years of rhGH treatment and 32 no GHD hypopituitary patients (19 F, 13 M, mean age 48.84 ± 15.55years, range 27–75 years) who achieved at least seven consecutive years of follow-up (**Figure 1**).

All GHD patients had known pituitary disease or other anterior pituitary hormonal deficiencies. GHD was mainly caused by treatment for pituitary adenomas (20 patients: 17 treated with surgery and three treated with surgery and radiotherapy (RT)). In 10 patients GHD was due to primary empty sella or pituitary hypoplasia and in four patients GHD occurred after treatment for craniopharyngioma (two treated with surgery and two treated with surgery and RT). Indeed, one patient had GHD for other pituitary lesions (arachnoid cyst treated with surgery). Finally, in two patients GHD occurred after traumatic brain injury and in two patients for Sheehan’s syndrome. (**Table 1**). Most GHD patients had multiple anterior pituitary hormonal deficiencies (**Table 2**). The diagnosis of GHD was based on a peak GH <9 µ/l after GHRH + Arginine (GHRH + ARG) test. All GHD patients were divided in two groups: group A, 10 EGHD patients (five F, five M, mean age 66.6 ± 2 years, range 65–71 years); group B, 29 AGHD patients (16 F, 13 M, mean age 42.1 ± 8.9 years, range 26–55 years) (**Figure 1**).

**Table 1:** Causes of pituitary deficiency in the study population of 39 GHD patients.

Causes	N of Patients
Pituitary adenoma	20
Primary empty sella/Pituitary hypoplasia	10
Craniopharyngioma	4
Other pituitary lesions (Arachnoid Cyst)	1
Traumatic brain injury	2
Sheehan’s Syndrome	2
Total	39

GHD, growth hormone deficiency.

**Table 2:** Number of pituitary deficiencies in the study population of 39 GHD patients.

Type of deficiency	N of Patients
Isolated GHD 1 additional deficiency	3 6
2 additional deficiencies	8
3 additional deficiencies	16
4 additional deficiencies (+ Diabetes insipidus)	6

GHD, growth hormone deficiency.

Causes of hypopituitarism in no GHD hypopituitary patients were: treatment for pituitary adenomas (17 patients: 15 treated with surgery and two treated with surgery and RT). In nine patients hypopituitarism was due to primary empty sella or pituitary hypoplasia, and in three patients hypopituitarism occurred after treatment for craniopharyngioma (two treated with surgery and one treated with surgery and RT). Indeed, one patient had GHD for other pituitary lesions (ependymoma treated with surgery and RT). Finally, in two patients GHD occurred after traumatic brain injury (**Table 3**). A number of anterior pituitary hormonal deficiencies are shown in **Table 4**. All no GHD hypopituitary patients were divided in two groups: group C, eight elderly no GHD hypopituitary patients (four F, four M, mean age 69.87 ± 3.31years, range 65–75 years); group D, 24 adult no GHD hypopituitary patients (15 F, nine M, mean age 41.83 ± 10.61 years, range 27–63 years) (**Figure 1**).

**Table 3:** Causes of pituitary deficiency in the study population of 32 no GHD hypopituitary patients.

Causes	N of Patients
Pituitary adenoma	17
Primary empty sella/Pituitary hypoplasia	9
Craniopharyngioma	3
Other pituitary lesions (ependymoma)	1
Traumatic brain injury	2
Total	32

GHD, growth hormone deficiency.

**Table 4:** Number of pituitary deficiencies in the study population of 32 no GHD hypopituitary patients.

Type of deficiency	N of Patients
1 deficiency	5
2 deficiencies	6
3 deficiencies	14
4 deficiencies (+ Diabetes insipidus)	7

GHD, growth hormone deficiency.

We recruited also 37 age-matched controls (21 F, 16 M, mean age 48.21 ± 16.29 years, range 26–72 years), which were divided in two groups: group E, nine elderly controls (five F, four M, mean age 67.77 ± 2.63 years, range 65–72 years); group F, 28 adult controls (16 F, 12 M, mean age 41.92 ± 13.53 years, range 26–64 years).

### Parameters

All parameters were evaluated at baseline and after three months during the first year of therapy and every six months during the following seven years of therapy.

In each group of GHD patients we evaluated body height, body weight, body mass index (BMI), waist circumference (WC), hip circumference (HC), waist–hip ratio (WHR), systolic blood pressure (SBP), and diastolic blood pressure (DBP). Body weight was measured in the morning to the nearest 0.1 kg, and body height was measured to the nearest 0.01 m while subject was wearing light indoor clothes without shoes. BMI was calculated as body weight (in kilograms) divided by squared height (in squared meters). WC was measured with a soft tape, midway between the lowest rib margin and the iliac crest, in the standing position. HC was measured over the widest part of the gluteal region, and then WHR was calculated.

BP was measured at the right arm with the patient in the sitting position. The average of three measurements using a mercury sphygmomanometer. Hypertension was diagnosed when SBP exceeded 140 mmHg, and DBP exceeded 90 mmHg.

The presence of MS was evaluated according to both the IDF and the ATPIII criteria. Thus, using the IDF criteria, the subject with abdominal obesity, defined as a WC >94 cm for men and WC >80 cm for woman, was considered to have MS, with at least two of the following factors: high values of TG value: >150 mg/dl or specific treatment for such dyslipidemia, reduced HDL-C values: <40 mg/dl in humans and <50 mg/dl in women or hypercholesterolemia, high BP >130/85 mm/Hg or hypertension treatment, high fasting blood glucose: >100 mg/dl or diagnosis of type 2 diabetes mellitus (17–19). Using the ATPIII criteria, subject was considered to have MS if subject had three of the following factors: abdominal obesity, defined as a WC >102 cm for men and WC >88 cm for women, high values of TG value: >150 mg/dl or specific treatment for such dyslipidemia, reduced HDL-C values: <40 mg/dl in men and <50 mg/dl in women or hypercholesterolemia, high BP >130/85 mm/Hg or hypertension treatment, high fasting blood glucose: >100 mg/dl or diagnosis of type 2 diabetes mellitus (20).

In each group of GHD patients we evaluated total cholesterol (TC), HDL-cholesterol (HDL-C), LDL-cholesterol (LDL-C), triglycerides (TG), blood glucose, glycosylated hemoglobin (HbA1c), and IGF-I; Venous blood sampling was performed between 8 and 12 am. The blood samples were immediately centrifuged, and the sera were stored at a temperature of −80°C until they were analyzed. Serum levels of TC, HDL-C, and TG were determined by enzymatic method on fasting serum. LDL-C was calculated according to Friedewald’s formula adjusted to SI units (21). Serum LDL-C was excluded in patients with serum TG >400 mg/dl. Blood glucose was measured by fasting on serum or plasma sample. Blood HbA1c was determined by HPLC method.

For the diagnosis of GHD, the GHRH + Arginine stimulation test was used, in agreement with studies in which adult patients with GHD with GH peak after ITT <3 μg/l have a GH response to GHRH + ARG <9 μg/l, while normal subjects have a GH response always >16.5 μg/l (22). Arginine (arginine hydrochloride, Damor, Naples, Italy and SALF^®^, Bergamo, Italy) was administered at a dose of 0.5 g/kg up to a maximum dose of 30 g by slow infusion from 0 to 30 min, while GHRH (Geref, Serono, Rome, Italy and GHRH Ferring, Milan, Italy) was injected at a dose of 1 μg/kg in intravenous bolus at time 0. Blood withdrawals were performed every 30 min from time 0 to 90 min.

Serum GH was measured by RIA using kits provided by Radim (Pomezia, Italy): the normal GH range was less than 5 ng/ml, and the sensitivity was 0.2 mg/L, and by CLIA using Liaison hGH kit of Diasorin: the hGH sensitivity is 0.052 μg/l; thus undetectable GH levels were arbitrarily considered 0.05 μg/l. The intraassay CVs were 4.4, 1.6, and 2.0% for the low, medium, and high points of the standard curve, respectively. The inter-assay CVs were 6.0, 7.7, and 6.8% for the low, medium, and high points of the standard curve. The hGH values were evaluated against the World Health Organization Second International Standard reference reagent 98/574, when possible. Plasma IGF-I was measured after ethanol extraction by immunoradiometric assay using kits provided by Diagnostic System Laboratories (Webster, TX): in our laboratory the normal IGF-I ranges in 20- to 30-, 31- to 40-, 41- to 50-, and over 50-year-old subjects were 110–502, 100–494, 100–303, and 78–258 ng/ml, respectively and by CLIA after automatized extraction using Liaison IGF-I kit of DiaSorin: The IGF-I sensitivity is <3 μg/l. The intra-assay CVs were 4.3, 3.0, and 3.3% for the low, medium, and high points of the standard curve, respectively. The inter-assay CVs were 4.4, 3.3, and 3.6% for the low, medium, and high points of the standard curve. The IGF-I values were evaluated against 1st WHO International Standard for Insulin-like Growth Factor-I NIBSC 02/254, when possible. In addition, an assessment of other pituitary axis hormones (TSH, FT3, FT4, FSH, LH, estradiol, testosterone, PRL, ACTH, cortisol, and urinary free cortisol) in order to determine the adequacy of substitution therapy, was periodically performed (at baseline, after three months, and every six months). Serum testosterone, estradiol, free forms of thyroid hormones, TSH, FSH, LH, PRL, and free urinary cortisol have been dosed with common kits on the market.

### Treatment Regimens

Each GHD patient initially received a GH dose according to regimens suggested by the international guidelines at the time of diagnosis (Group A, mean dose: 1.5 ± 0.2 mg/week; group B, mean dose: 2 ± 0.7 mg/week). During the following years of treatment, the GH dose was gradually titrated on the basis of IGF-I concentration (according to age/sex related values) (23). Dose titration and safety monitoring were performed every three months during the first year and every six months thereafter. When required, in GHD and no GHD hypopituitary group, patients received adequate and stable therapy with glucocorticoids, thyroid hormone, testosterone, estrogen, and desmopressin.

### Statistical Analysis

Data were analyzed using SPSS Software for Windows, version 20.0 (SPSS, Inc., Cary, NC package) and were reported as Mean ± Standard Deviation (SD) or as percentages. For all variables, within-group differences were calculated using a repeated-measures ANOVA, followed by a *post hoc* analysis performed using Bonferroni or Student–Newman–Keuls tests where applicable. The t student test was used for intergroup/intragroup comparison. Then the prevalence of arterial hypertension, dyslipidemia, MS according to both IDF and ATPIII criteria and DM was calculated for evaluation of intragroup differences with the chi-square test. Significance was set at 5%.

## Results

### GH Dose, Serum IGF-I, Body Composition, and Blood Pressure

Mean rhGH dose at baseline was 1.52 ± 0.26 mg/week in Group A and 2.02 ± 0.74 mg/week in group B. During follow-up rhGH dose was gradually titrated according IGF-I normal value for age and sex with a mean dose of 0.96 ± 0.46 mg/week in Group A and 3.07 ± 2.28/week in Group B after seven years’ treatment. Serum IGF-I concentration was increased in the two groups with a mean value within the normal range (± 2 S.D.) during the follow-up. After seven years of therapy, WC and HC significantly decreased in Group B (p = 0.004 and p = 0.011 respectively). WHR significantly decreased in Group B (p = 0.021). In group A there was a significant reduction only of HC (p = 0.03) with no modification of WC and WHR. No differences in Group A and B were found in BP parameters (**Tables 5** and **6**). No differences in these parameters were observed between groups, except for SBP that was higher in Group A than in Group B (p <0.001) (**Table 7**).

**Table 5:** Clinical features of EGHD (Group A) patients at baseline and after 7 years of rhGH treatment.

Parameters	Baseline	7 years	P
BW (kg)	74.18 ± 11.72	73.40 ± 12.39	NS
BMI (kg/m^2^)	30.44 ± 3.87	30.21 ± 4.18	NS
WC (cm)	99.30 ± 6.01	98.50 ± 8.19	NS
HC (cm)	106.10 ± 3.66	102.70 ± 5.49	0.03
WHR	0.93 ± 0.02	0.95 ± 0.03	NS
SBP (mmHg)	139.20 ± 6.56	130.9 ± 13.56	NS
DBP (mmHg)	75.30 ± 11.77	79.60 ± 9.30	NS
TC (mg/dL)	219.50 ± 25.84	193.40 ± 42.91	NS
LDL-C (mg/dl)	125.96 ± 24.56	112.38 ± 19.55	NS
HDL-C (mg/dl)	52 ± 7.21	53.10 ± 11.14	NS
TG (mg/dl)	206.70 ± 96.18	139.5 ± 34.2	0.048
Fasting glucose (mg/dl)	87.80 ± 13.54	88.80 ± 6.21	NS
HbA1c (%)	5.69 ± 0.63	5.75 ± 0.59	NS
IGF-I (mg/dl)	75.64 ± 35.18	165.80 ± 78.00	0.002
GH dose (mg/week)	1.52 ± 0.26	0.96 ± 0.46	0.009

EGHD, elderly growth hormone deficiency; rhGH, recombinant human growth hormone; BW, body weight; BMI, body mass index; WC, waist circumference; HC, hip circumference; WHR, waist to hip ratio, SBP, systolic blood pressure; DBP, diastolic blood pressure; TC, total cholesterol; LDL-C, low density lipoprotein cholesterol; HDL-C, high density lipoprotein cholesterol; TG, triglycerides; HbA1c, glycated haemoglobin.

**Table 6:** Clinical features of AGHD (Group B) patients at baseline and after 7 years of rhGH treatment.

Parameters	Baseline	7 years	P
BW (kg)	77.73 ± 16.92	79.01 ± 19.49	NS
BMI (kg/m^2^)	29.24 ± 5.68	29.60 ± 6.00	NS
WC (cm)	105.72 ± 14.60	94.27 ± 14.11	0.004
HC (cm)	110.98 ± 10.19	104.43 ± 8.74	0.011
WHR	0.95 ± 0.08	0.90 ± 0.08	0.021
SBP (mmHg)	121.24 ± 14.19	123.00 ± 18.00	NS
DBP (mmHg)	76.10 ± 8.61	77.52 ± 10.4	NS
TC (mg/dl)	230.9 ± 41.35	200.18 ± 39.7	0.006
LDL-C (mg/dl)	149.47 ± 35.71	116.60 ± 21.06	<0.0001
HDL-C (mg/dl)	50.17 ± 15.57	60.01 ± 15.80	0.020
TG (mg/dl)	154.28 ± 92.06	116.62 ± 39.06	0.047
Fasting glucose (mg/dl)	84.79 ± 15.99	90.83 ± 21.84	0.023
HbA1c (%)	5.73 ± 0.65	5.61 ± 0.73	NS
IGF-I (mg/dl)	84.25 ± 41.57	178.94 ± 80.17	<0.001
GH dose (mg/week)	2.02 ± 0.74	3.07 ± 2.28	0.016

AGHD, adult growth hormone deficiency; rhGH, recombinant human growth hormone; BW, body weight; BMI, body mass index; WC, waist circumference; HC, hip circumference; WHR, waist to hip ratio, SBP, systolic blood pressure; DBP, diastolic blood pressure; TC, total cholesterol; LDL-C, low density lipoprotein cholesterol; HDL-C, high density lipoprotein cholesterol; TG, triglycerides; HbA1c, glycated haemoglobin.

**Table 7:** Comparison between Group A (EGHD) and Group B (AGHD) patients at baseline and after 7 years of rhGH treatment.

Parameters	Baseline	7 years
	P Group A *vs* B	P Group A *vs* B
BW	NS	NS
BMI	NS	NS
WC	NS	NS
HC	NS	NS
WHR	NS	NS
SBP	<0.001^a^	NS
DBP	NS	NS
TC	NS	NS
LDL-C	NS	NS
HDL-C	NS	NS
TG	NS	NS
Fasting glucose	NS	NS
HbA1c	NS	NS
IGF-I	NS	NS
GH dose	NS	NS

^a^A > B. EGHD, elderly growth hormone deficiency; AGHD, adult growth hormone deficiency; rhGH, recombinant human growth hormone; BW, body weight; BMI, body mass index; WC, waist circumference; HC, hip circumference; WHR, waist to hip ratio, SBP, systolic blood pressure; DBP, diastolic blood pressure; TC, total cholesterol; LDL-C, low density lipoprotein cholesterol; HDL-C, high density lipoprotein cholesterol; TG, triglycerides; HbA1c, glycated haemoglobin. NB, data of parameters of each group are shown in **Tables 5** and **6**.

### Lipid Profile and Glucose Metabolism

TC significantly decreased in Group B (p = 0.006) after seven years of treatment. In Group B also HDL-C increased (p = 0.02), and LDL-C (p <0.001) significantly decreased at study end. Indeed, TG significantly decreased in Groups A and B (p = 0.048 and p = 0.047 respectively) (**Tables 5** and **6**). No differences were observed in other lipid parameters between groups at baseline and after treatment (**Table 7**).

Blood glucose increased in Group B (p = 0.023), while there was no difference in HbA1c after seven years of rhGH (**Tables 5** and **6**). No significant modifications of these parameters were found between groups at baseline and at study end (**Table 7**).

### Prevalence of Dyslipidemia, Metabolic Syndrome, Diabetes Mellitus, and Hypertension

In Group B, an increased prevalence of hypertension (13.7 *vs* 90%) was observed (p = 0.031). No differences were observed on prevalence of hypertension in Group A and on prevalence of dyslipidemia, MS-IDF, MS-ATP, and DM in Groups A and B (**Table 8**). There was no difference in the prevalence of all these comorbidities in Groups C and D (**Table 9**). In Groups E and F there was a higher prevalence of dyslipidemia (p = 0.045 and p = 0.034 respectively) after 7 years of follow-up (**Table 10**).

**Table 8:** The prevalence of metabolic co-morbidities at study entry and after 7 years of rhGH treatment in each group of GHD patients (elderly and adult patients).

Patients		Dyslipidemia n (%)	MS-IDF n (%)	MS-ATPIII n (%)	DM n (%)	Arterial hypertension n (%)
**Group A**	**Baseline**	7 (70)	7 (70)	6 (60)	1 (10)	9 (90)
**(10 patients)**	**7 years**	7 (70)	7 (70)	7 (70)	1 (10)	8 (80)
	**p**	NS	NS	NS	NS	NS
**Group B**	**Baseline**	15 (51.7)	7 (17.2)	3(10.3)	2 (6.89)	4 (13.7)
**(29 patients)**	**7 years**	16 (55.1)	6 (20.6)	4 (13.7)	3 (10.3)	8 (27.5)
	**p**	NS	NS	NS	NS	0.031

Group A, elderly growth hormone deficiency patients; Group B, adult growth hormone deficiency patients; rhGH, recombinant human growth hormone deficiency; MS-IDF, metabolic syndrome according IDF criteria; MS-ATPIII, metabolic syndrome according ATPIII criteria; DM, diabetes mellitus.

**Table 9:** The prevalence of metabolic co-morbidities at study entry and after 7 years in each group of no GHD hypopituitary patients (elderly and adult patients).

Patients		Dyslipidemia n (%)	MS-IDF n (%)	MS-ATPIII n (%)	DM n (%)	Arterial hypertension n (%)
**Group C**	**Baseline**	4 (50)	2 (25)	2 (25)	2 (25)	4 (50)
**(8 patients)**	**7 years**	4 (50)	4 (50)	4 (50)	2 (25)	4 (50)
	**p**	NS	NS	NS	NS	NS
**Group D**	**Baseline**	11 (45.8)	8 (33.3)	6 (25)	2 (8.33)	8 (33.3)
**(24 patients)**	**7 years**	7 (29.1)	10 (41.6)	8 (33.3)	2 (8.33)	7 (29.1)
	**p**	NS	NS	NS	NS	NS

Group C, elderly no GHD hypopituitary patients; Group D, adult no GHD hypopituitary patients; MS-IDF, metabolic syndrome according IDF criteria; MS-ATPIII, metabolic syndrome according ATPIII criteria; DM, diabetes mellitus.

**Table 10:** The prevalence of metabolic co-morbidities at study entry and after 7 years in each group of controls (elderly and adult controls).

Patients		Dyslipidemia n (%)	MS-IDF n (%)	MS-ATPIII n (%)	DM n (%)	Arterial hypertension n (%)
**Group E**	**Baseline**	4 (44.4)	3 (33.3)	3 (33.3)	2 (22.2)	4 (44.4)
**(9 patients)**	**7 years**	8 (88.8)	4 (44.4)	3 (33.3)	2 (22.2)	5 (55.5)
	**p**	0.045	NS	NS	NS	NS
**Group F**	**Baseline**	17 (60.7)	8 (28.5)	9 (32.1)	4 (14.2)	4 (14.2)
**(28 patients)**	**7 years**	24 (85.7)	9 (32.1)	10 (35.7)	4 (14.2)	8 (28.5)
	**p**	0.034	NS	NS	NS	NS

Group E, elderly controls; Group F, adult controls; MS-IDF, metabolic syndrome according IDF criteria; MS-ATPIII, metabolic syndrome according ATPIII criteria; DM, diabetes mellitus.

We also compared the prevalence of dyslipidemia, MS, DM, and hypertension of all our GHD patients, according to age, to prevalence of these diseases in no GHD hypopituitary patients and in controls. There were no differences between GHD patients (Groups A and B) and no GHD hypopituitary patients (Groups C and D), but there was a higher prevalence of dyslipidemia in adult controls (Group F) than adult GHD (Group B) after 7 years (p = 0.011) (**Tables 11** and **12**).

**Table 11:** Differences in prevalence of metabolic co-morbidities at study entry and after 7 years between the two groups of elderly patients (GHD and no GHD hypopituitary patients) and between elderly GHD patients and elderly controls.

	Patients	Dyslipidemia n (%)	MS-IDF n (%)	MS-ATPIII n (%)	DM n (%)	Arterial hypertension n (%)
**Baseline**	**A**	7 (70)	7 (70)	6 (60)	1 (10)	9 (90)
	**C**	4 (50)	2 (25)	2 (25)	2 (25)	4 (50)
	**p**	NS	NS	NS	NS	NS
**7 years**	**A**	7 (70)	7 (70)	7 (70)	1 (10)	8 (80)
	**C**	4 (50)	4 (50)	4 (50)	2 (25)	4 (50)
	**p**	NS	NS	NS	NS	NS
						
**Baseline**	**A**	7 (70)	7 (70)	6 (60)	1 (10)	9 (90)
	**E**	4 (44.4)	3 (33.3)	3 (33.3)	2 (22.2)	4 (44.4)
	**p**	NS	NS	NS	NS	NS
**7 years**	**A**	7 (70)	7 (70)	7 (70)	1 (10)	8 (80)
	**E**	8 (88.8)	4 (44.4)	3 (33.3)	2 (22.2)	5 (55.5)
	**p**	NS	NS	NS	NS	NS

Group A, elderly GHD patients; Group C, elderly no GHD hypopituitary patients; Group E, elderly controls; MS-IDF, metabolic syndrome according IDF criteria; MS-ATPIII, metabolic syndrome according ATPIII criteria; DM, diabetes mellitus.

**Table 12:** Differences in prevalence of metabolic co-morbidities at study entry and after 7 years between the two groups of adult patients (GHD and no GHD hypopituitary patients) and between adult GHD patients and elderly controls.

	Patients	Dyslipidemia n (%)	MS-IDF n (%)	MS-ATPIII n (%)	DM n (%)	Arterial hypertension n (%)
**Baseline**	**B**	15 (51.7)	7 (17.2)	3(10.3)	2 (6.89)	4 (13.7)
	**D**	11 (45.8)	8 (33.3)	6 (25)	2 (8.33)	8 (33.3)
	**p**	NS	NS	NS	NS	NS
**7 years**	**B**	16 (55.1)	6 (20.6)	4 (13.7)	3 (10.3)	8 (27.5)
	**D**	7 (29.1)	10 (41.6)	8 (33.3)	2 (8.33)	7 (29.1)
	**p**	NS	NS	NS	NS	NS
						
**Baseline**	**B**	15 (51.7)	7 (17.2)	3(10.3)	2 (6.89)	4 (13.7)
	**F**	17 (60.7)	8 (28.5)	9 (32.1)	4 (14.2)	4 (14.2)
	**p**	NS	NS	NS	NS	NS
**7 years**	**B**	16 (55.1)	6 (20.6)	4 (13.7)	3 (10.3)	8 (27.5)
	**F**	24 (85.7)	9 (32.1)	10 (35.7)	4 (14.2)	8 (28.5)
	**p**	0.011	NS	NS	NS	NS

Group B, adult GHD patients; Group D, adult no GHD hypopituitary patients; Group F, adult controls; MS-IDF, metabolic syndrome according IDF criteria; MS-ATPIII, metabolic syndrome according ATPIII criteria; DM, diabetes mellitus.

## Discussion

This is a single-center observational study on the effects of long-term rhGH replacement on body composition and cardiovascular risk factors in GHD patients, dividing subjects in EGHD and AGHD patients according to the age of onset of GHD. As there are limited data in literature on the long-term effect of GH therapy in elderly patients, our paper implements and strengthens the knowledge on the long-term management of rhGH therapy, with particular regard to its efficacy and safety, in a subset of patients in whom GHD contributes to their fragility. In addition, although there are other similar studies with matched populations, these studies have shorter follow-up periods or, if long enough, to evaluate GHD adult patients without differences between adult and elderly people.

In our study 7 years of GH treatment improved body composition of AGHD patients. In fact, in our AGHD patients, WC, HC, and WHR decreased after GH therapy, and this is in line with other short-term studies (24, 25). Even if we did not perform a body composition analysis with DEXA or magnetic resonance, we used WHR as indicator of visceral fat mass (VFM) according to the World Health Organization (WHO) that states that abdominal obesity is defined as a WHR above 0.90 for M and above 0.85 for F (26). Indeed, WHR is reported to be of clinical utility in identifying patients with cardiovascular risk factors in an adult population (27–29). WC is also a parameter used as indicator of VFM (30), and in our AGHD patients WC was decreased. So, the reduction in WC and WHR in our AGHD patients should reflect an effect of GH to limit central body fat deposit that occurs with age (31) and suggest a reduction in VFM in our patient. This is in line with a previous study of Svensson et al. in which seven years of GH treatment reduced body fat (32). These changes are reported also in longer observational studies (≥10 years) (33, 34) where body fat was reduced when corrected for age related-increase in body fat in normal aging (35). Using Framingham model, the GH-induced difference in WHR with an improvement in body composition would represent a 3–4% decrease in the incidence of coronary heart disease over 10 years (36, 37).

In our EGHD patients there was only a reduction of HC, while WC and WHR were not modified by GH treatment, and this is in contrast with long term (10 years) follow-up results study (38) in which authors assessed a reduction in body fat (BF) (especially after 5 years of treatment). This difference probably reflects the use of a different technique (DEXA) which is a direct method of body composition measurement and the slightly younger mean age of the Swedish study’s patients, who would be less affected than our patients by the increased central body fat that occurs with aging (35). Other studies about body composition and metabolic parameters in EGHD have a less treatment period (≤2 years) (16, 39–43).

Regarding lipid profile, in our AGHD patients TC and LDL-C were decreased and HDL-C was increased. This pattern did not change after exclusion of patients using lipid-lowering drugs and is due not only to improvements in body composition, but also to a direct effect of GH on lipid metabolism. In fact, GH increases the expression of LDL receptors (44), which increases the clearance of LDL-C as well as the hepatic uptake of partially de-lipidated VLDL particles (45). GH also enhances the available intrahepatic lipid substrate through its lipolytic action, stimulating very low-density lipoprotein (VLDL) apo B secretion (44). Other long-term studies showed an improvement in lipid profile after GH substitution (32–34, 46–50) with a reduction in TC and LDL-C and an increase in HDL-C. In the general population, the cardiovascular risk of hypercholesterolemic patients is reduced to 15% by the reduction in 10% cholesterol levels (51), so any additional effect of GH treatment next to conventional lipid-lowering drugs may be advantageous.

In contrast to our findings, the majority of the mentioned studies did not report a reduction in TG that occurred in our cohort, except for the observational study of Gotherstrom and colleagues (49) in which there was a reduction of TG after 5 years of GH replacement. This finding was not confirmed by the same Sweden study group after 10 (34) and 15 (33) years of therapy. GH activates the hormone-sensitive lipase, increases triglycerides hydrolysis in fatty acids and glycerol, induces lipolysis, and reduces the re-esterification of free fatty acids to triglycerides (52). These effects can explain our findings. EGHD patients showed no changes in lipid profile except for a reduction in TG; TC and LDL-C had a decreasing trend and HDL-C an increasing one even if not significant. Previous studies (39, 41–43, 53) reported in EGHD lipid changes similar to AGHD patients.

Despite favorable changes in lipid profile, the prevalence of dyslipidemia does not decrease after therapy in the two groups of our patients, but the reduction of LDL-C levels after treatment is of particular importance if we consider the correlation between LDL-C blood levels and cardiovascular risk (54–56). Comparing the prevalence of dyslipidemia of our patients, according to age, to prevalence of this comorbidity in no GHD hypopituitary patients and in controls, we found no differences between GHD patients and no GHD hypopituitary patients, but there was a higher prevalence of dyslipidemia in adult controls than adult GHD patients after 7 years. Probably, this is due to the fact that in the control group there was a significant increase of prevalence of dyslipidemia after 7 years of follow-up, both in elderly and adult.

About glucose metabolism, as demonstrated in previous studies (33, 34, 49, 57, 58) we observed in our AGHD cohort an increase in blood glucose, even if there was no modification in HbA1c after seven years of rhGH treatment. The increase in fasting blood glucose concentration could, at least to some extent, be an effect of the normal aging of patients. It was hypothesized by Gotherstrom and colleagues (49) that this increase in fasting blood glucose in the morning may be affected by the bedtime GH injections and does not reflect 24-h glucose homeostasis in GHD adults receiving GH replacement which is lower (59). This is in line with no modification of HbA1c that is observed in our study and that is the expression of the mean blood glucose level during several weeks.

The insulin sensitivity impairs with increasing age in the general population (60, 61). It is known that during initial months of GH treatment, there is an initial deterioration of insulin sensitivity that could return to baseline values after several months of treatment, probably for the favorable effect of GH on body composition (49) and on the well-being and physical activity. In a study of Christopher and colleagues (62), authors found unchanged insulin sensitivity after 2 years of GH treatment compared with baseline. In another long-term study insulin sensitivity was determined using hyperinsulinemic, euglycemic clamp technique in 11 GHD patients after 7 years of treatment (32). The authors reported an initial increase of blood glucose during the first year of treatment with an unchanged insulin sensitivity at study end even if there was a tendency to be higher than in controls. Since in our GHD patient we have no modification of HbA1c and since it is known that there is an inverse correlation between insulin sensitivity and TG (63), the reduction of TG observed in our study may imply an improvement in insulin sensitivity in our patients. RhGH therapy could therefore prevent the age-related insulin sensitivity decline in treated GHD patients.

Our EGHD patients showed no change in blood glucose and HbA1c levels. This is in line with results of another study (64) in which GH therapy did not affect glucose metabolism, although the follow-up is too short (1 years).

All groups of GHD patients of our study showed no modification of prevalence of DM at the end of follow-up, and this prevalence did not differ from the prevalence of DM in no GHD hypopituitary patients and in controls at study end.

All patients who had DM in our study were obese (BMI ≥30 kg/m^2^). This is confirmed in other two studies based on international database (65, 66) where obesity and disturbed glucose metabolism at baseline increased risk of DM whose incidence was reported similar to the background population (66). In a more recent study using Patro database (67), the authors conclude that rhGH treatment did not increase the risk of DM and glucose impairments.

Concerning BP, contrasting with two long term studies (33, 57) that reported higher SBP after treatment, in our EGHD and AGHD patients we found no difference in SBP and DBP after 7 years’ treatment, even if we observed an increase in prevalence of hypertension in AGHD.

Moreover, in our EGHD patients SBP was found higher than AGHD. These two findings may reflect the increase in BP with normal aging. Despite these results, we found no difference between the prevalence of hypertension in GHD patients and the prevalence of hypertension in no GHD hypopituitary patients and in controls.

Regarding the prevalence of MS-IDF and MS-ATPIII, there were no modifications in both GHD groups after GH treatment, even if there was a not significant trend to be higher at the end of the study according to the ATPIII criteria. Despite improvement of lipid spectrum, this increased MS-ATPIII prevalence probably is mainly due to an increase in BP and hyperglycemia which was reported respectively in EGHD and AGHD patients. Two studies have shown a persistently high MS prevalence after 3 and 5 years of rhGH treatment, respectively (50, 68). In the study of Claessen et al (48)., the authors found a further increase in MS prevalence to 57% after 10 years of rhGH substitution. However, in our study we found no differences if this prevalence is compared to the prevalence of MS in no GHD hypopituitary patients and in controls.

A limitation of our study is the lack of a control GHD group and thereby the lack of control for aging. Because the beneficial effects of rhGH therapy are well-established in the short term, it is unethical to deny GH therapy for long period to patients with GHD when no contraindications were assessed. Therefore, it is difficult if not impossible, to perform long-term randomized, controlled trials including GHD patients without rhGH treatment.

To reduce the effect of this limitation on our assessments, we compared our results with data of no GHD hypopituitary patients and healthy controls.

Another limitation is the relative small number of patients in the EGHD cohort, but it was difficult for us to recruit a large number of these patients; some of them stopped GH treatment before the established follow-up period for lack of subjective improvement or opted not to have GH therapy because they disliked injections or because they felt sufficiently well without additional treatment.

In conclusion, the present study showed the beneficial effects of long-term rhGH therapy in GHD patients on body composition and lipid profile as in line with other previous studies. These effects are more evident in AGHD than EGHD patients. The improvement in lipid profile is confirmed by finding that adult GHD patients have lower prevalence of dyslipidemia than controls. Findings on glucose metabolism are contrasting although the prevalence of DM type 2 is similar to controls. The increases in glucose levels, BMI, and SBP in GHD negatively affected the prevalence of the MS in the long term, especially in AGHD patients even if there is no difference of MS prevalence with control population.

Future larger studies are needed to investigate whether the increased mortality seen in hypopituitary GHD adults not receiving GH can be affected by GH replacement, especially in EGHD patients in which an analysis including cost-effectiveness and QoL is required.

## Data Availability Statement

The original contributions presented in the study are included in the article/supplementary material. Further inquiries can be directed to the corresponding author.

## Ethics Statement

The studies involving human participants were reviewed and approved by the Dipartimento di Scienze Biomediche Avanzate-Sezione di Medicina Legale-Università di Napoli “Federico II”, Naples Italy. The patients/participants provided their written informed consent to participate in this study.

## Author Contributions

The authors’ responsibilities were as follows: ES and CD were responsible for the concept of this paper and drafted the manuscript. ER, TS, RA, FR, ED, GD, and AC provided a critical review of the paper. All authors contributed to the article and approved the submitted version.

## Conflict of Interest

The authors declare that the research was conducted in the absence of any commercial or financial relationships that could be construed as a potential conflict of interest.
